# Identification of transcription factors interacting with a 1274 bp promoter of MaPIP1;1 which confers high-level gene expression and drought stress Inducibility in transgenic *Arabidopsis thaliana*

**DOI:** 10.1186/s12870-020-02472-7

**Published:** 2020-06-16

**Authors:** Yi Xu, Zhiqiang Jin, Biyu Xu, Jingyang Li, Yujia Li, Xiaoyi Wang, Anbang Wang, Wei Hu, Dongmei Huang, Qing Wei, Zhuye Xu, Shun Song

**Affiliations:** 1 Key Laboratory of Genetic Improvement of Bananas, Haikou Experimental Station, Chinese Academy of Tropical Agricultural Sciences, Haikou, China; 2 Key Laboratory of Tropical Crop Biotechnology, Institute of Tropical Bioscience and Biotechnology, Chinese Academy of Tropical Agricultural Sciences, Haikou, China; 3 Hainan University, Haikou, China

**Keywords:** Aquaporin, Promoter, Droutht stress, 5′ deletion, Banana, Transcription factor

## Abstract

**Background:**

Drought stress can severely affect plant growth and crop yield. The cloning and identification of drought-inducible promoters would be of value for genetically-based strategies to improve resistance of crops to drought.

**Results:**

Previous studies showed that the MaPIP1;1 gene encoding an aquaporin is involved in the plant drought stress response. In this study, the promoter pMaPIP1;1, which lies 1362 bp upstream of the MaPIP1;1 transcriptional initiation site, was isolated from the banana genome..And the transcription start site(A) is 47 bp before the ATG. To functionally validate the promoter, various lengths of pMaPIP1;1 were deleted and fused to GUS to generate pMaPIP1;1::GUS fusion constructs that were then transformed into Arabidopsis to generate four transformants termed M-P1, M-P2, M-P3 and M-P4.Mannitol treatment was used to simulate drought conditions. All four transformants reacted well to mannitol treatment. M-P2 (− 1274 bp to − 1) showed the highest transcriptional activity among all transgenic Arabidopsis tissues, indicating that M-P2 was the core region of pMaPIP1;1. This region of the promoter also confers high levels of gene expression in response to mannitol treatment. Using M-P2 as a yeast one-hybrid bait, 23 different transcription factors or genes that interacted with MaPIP1;1 were screened. In an dual luciferase assay for complementarity verification, the transcription factor MADS3 positively regulated MaPIP1;1 transcription when combined with the banana promoter. qRT-PCR showed that MADS3 expression was similar in banana leaves and roots under drought stress. In banana plants grown in 45% soil moisture to mimic drought stress, MaPIP1;1 expression was maximized, which further demonstrated that the MADS3 transcription factor can synergize with MaPIP1;1.

**Conclusions:**

Together our results revealed that MaPIP1;1 mediates molecular mechanisms associated with drought responses in banana, and will expand our understanding of how AQP gene expression is regulated. The findings lay a foundation for genetic improvement of banana drought resistance.

## Background

Water is essential for plant growth and development [1]. Abiotic stress such as drought can affect plant growth, leading to major losses in plant production worldwide [2], and is a growing problem in agriculture [3–6]. All forms of water transport including drought adjustment, single cell expansion and long distance transport require movement of water through the cell membrane [7]. Plant aquaporins (AQPs) allow water influx and efflux to enhance water permeability in vacuolar and plasma membranes [8, 9]. AQPs play an important role in the physiology of water balance and promote efficient water use [10–14]. Water homeostasis is fundamental for cell survival. Transport of water across cellular membranes is governed by AQPs-tetrameric integral membrane channels that are highly conserved throughout the eukaryotic kingdoms [15]. In eukaryotes,it allows water flow across cellular membranes to be tightly regulated in response to various external and internal signals, thereby maintaining the desired water balance of the organism [16]. Fast regulation can be achieved by two means—either by altering the water permeability rate through the pore itself (gating) or by rapidly changing the abundance of AQP molecules in the plasma membrane by shuttling the protein between the plasma membrane and intracellular vesicles, so-called trafficking [17].

AQPs are transmembrane proteins, which form channels in intracellular and plasma membranes to facilitate rapid movement of water in either direction [18]. In order to transport water, some major intrinsic protein (MIP) family members can also transport glycerol, CO_2_, urea,ammonia, hydrogen peroxide, boron, silicon, arsenite, antimonite, lactic acid [19, 20] and O_2_ [21].

The large and highly diverse family of plant AQPs can be divided into 8 sub-families: plasma membrane intrinsic proteins (PIPs), tonoplast intrinsic proteins (TIPs), nodulin 26-like intrinsic proteins (NIPs), small basic intrinsic proteins (SIPs), GlpF-like intrinsic proteins (GIPs), hybrid intrinsic proteins (HIPs), uncategorized members designated X intrinsic proteins (XIPs), and large intrinsic proteins (LIPs) [22, 23]. Together these sub-families are involved in plant growth and development processes such as seed germination and fruit ripening, as well as cell elongation [24]. Moreover, AQPs play a central role in maintaining water homeostasis in plant responses to environmental stress [18, 25]. In recent years, AQPs have gained increasing interest for studies to understand how plants respond to abiotic stress. Many experimental results indicate that plants regulate AQP activity to respond to various abiotic stresses such as cold damage, salt damage, mechanical damage, drought stress, and heavy metal stress. In particular, AQPs play crucial roles in the response of plants to drought stress as evidenced by studies showing that overexpression of some AQP family genes in plants increases the tolerance of transgenic plants to drought stress [24–27]. For example, TaAQP7 overexpression increases tolerance of transgenic tobacco to drought stress [27]. Transformation of Arabidopsis with wheat TaTIP2;2 increased the tolerance of the resulting transgenic plants to drought stress [28]. In bananas, transgenic AQP genes can also improve drought resistance. Banana plants overexpressing MusaPIP1;2 showed significantly enhanced drought resistance compared to control plants [29]. In a study by Sreedharan, transgenic banana plants transformed with MusaPIP1;2 had more robust recovery after rehydration following drought treatment relative to wild type plants. Comparison of physiological and biochemical indices of transgenic and wild type plants showed that MusaPIP1;2-transformed plants had increased amounts of malondialdehyde with decreased proline and relative water content, indicating that the transgenic plants had enhanced ability to induce drought adjustments that translated to better drought resistance. Similarly, Arabidopsis transgenic for MaPIP1;1 also showed improved plant drought resistance [30].

Despite these findings, the molecular mechanisms that govern drought tolerance remain unclear. Abiotic stresses such as drought significantly affect plant growth and development as well as metabolism [31, 32]. Plants have multiple pathways to adapt rapidly to multiple stresses that promote survival under drought stress. A series of functional proteins are important for these response mechanisms such as molecular chaperones, osmoregulatory proteins, channel proteins, transport proteins, protective proteins and detoxification proteins. The expression of these functional proteins is controlled by specific transcription factors that interact with cis-acting elements to induce expression of specific genes in response to drought stress. Gene expression is quantitatively regulated by specific promoters that can contain multiple cis-regulatory elements [33]. Interactions between cis-elements and transcription factors (TFs) have a key role in activating or suppressing expression of target genes by coordinated regulation of transcription [34, 35]. At present,few promoters that can be used for genetic improvement of crop plants have been described. The most common type of promoter in plants is the 35 s promoter CaMV35S, which can drive high levels of gene expression in dicots, whereas the maize ubiquitin promoter drives gene expression in monocots. These promoters are capable of driving high levels of transgene expression in markers or monocots in almost all tissues and developmental stages [36–38]. Inducible or tissue-specific promoters modulate target gene expression under particular conditions or in certain tissues. To date, some tissue-specific and stress-inducible promoters have been reported for plants. For example, the rd29A and rd29B promoters in Arabidopsis respond to high salinity and drought [39]. In wheat, the Dreb2 promoter responds to drought stress [40]. The BjSOS2 gene promoter from mustard plant functions in response to high salt, drought and abiotic stresses as well as other forms of stress [5]. The durum wheat gene TdPIP2;1 and its promoter region are in response to abiotic stress in rice [41].

In this study, the promoter for the MaPIP1;1 gene was cloned, we divided the promoter into four parts, M-P1-M-P4, and transformed it into *Arabidopsis thaliana*. GUS staining and GUS inactivity assay showed that M-P2 is the core region of the promoter. A yeast one-hybrid method was used to identify transcription factors that directly regulate the MaPIP1;1 promoter, and molecular mechanisms by which target transcription factors modulate MaPIP1;1 expression. According to the cis-acting element, a 5′ deletion mutant of the promoter was used to determine the core region, and as bait in a yeast single-hybrid assay to identify 23 different transcription factors or genes that interact with MaPIP1;1. The transcription factor MADS3 was selected based on its binding results in an dual luciferase assay. Under 45% soil moisture to induce drought stress, MaPIP1;1 expression was maximized, thus demonstrating that MADS3 transcription factor cooperated with MaPIP1;1. Taken together, the results of this study revealed that MaPIP1;1 mediates the molecular regulation mechanism of drought stress responses in bananas, and will expand our understanding of how AQP gene expression is regulated in bananas exposed to drought stress. The findings lay a foundation for design of genetic modifications to improve banana drought resistance.

## Results

### Identification of MaPIP1;1 promoter and cis-acting element analysis

**Fig. 1:**
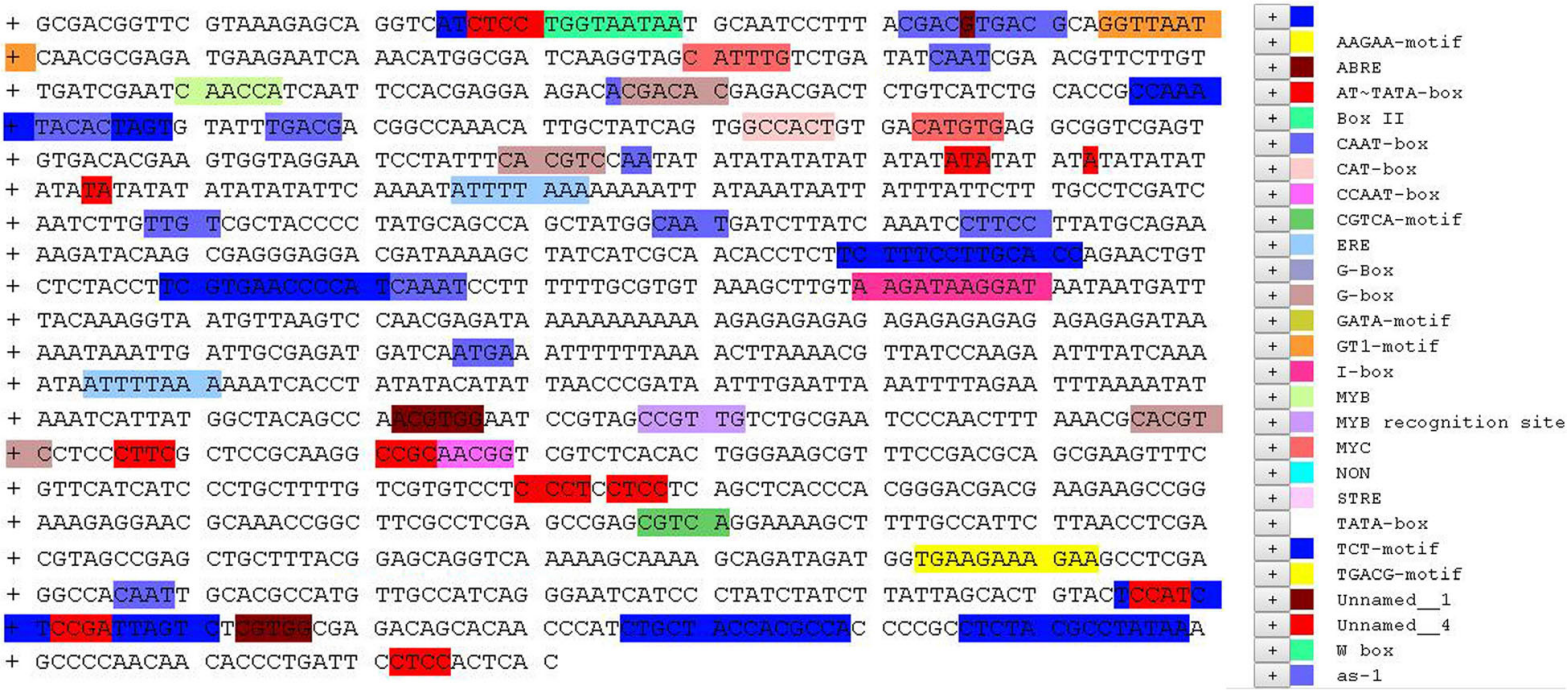
The 1362 bp promoter and the cis-acting element of MaPIP1;1. known cis-acting elements are shown in the right. The descriptions of elements see Table 1

The MaPIP1;1 promoter sequence was obtained from the banana A genome website (http://banana-genome.cirad.fr) and a specific primer was designed to amplify a 1362 bp fragment surrounding the promoter (Table 1), which is upstream of the MaPIP1;1 transcriptional initiation site. And the transcription start site(A) is 47 bp before the ATG. Analysis of the promoter sequence for putative cis-acting elements using PlantCARE and PLACE databases showed that this fragment contained multiple TATA-box and CAAT-box core cis-acting elements, an abscisic acid responsive element (ABRE), MYB element (CAACCA), two types of MYC elements (CATGTG and CATTTG), an ERE element (ATTTTAAA), MYB recognition site (CCGTTG), an AAGAA motif (TGAAGAAAGAA), a MYBHv1 binding site (CCAAT box), two types of MeJA (methyl jasmonate) responsive elements (TGACG-motif and CGTCA-motif), four types of light responsive elements (Box II,G-box, GT1-motif, I-BOX), a meristem responsive element (CAT-box), CCAAT box (CAACGG), MYB recognition site (CCGTTG), and an unknown element (CGTGA) (Fig. 1). Although MaPIP1;1 was induced by mannitol treatment, the pMaPIP1;1 sequence lacked known drought stress-inducible cis-acting elements.

**Table 1:**
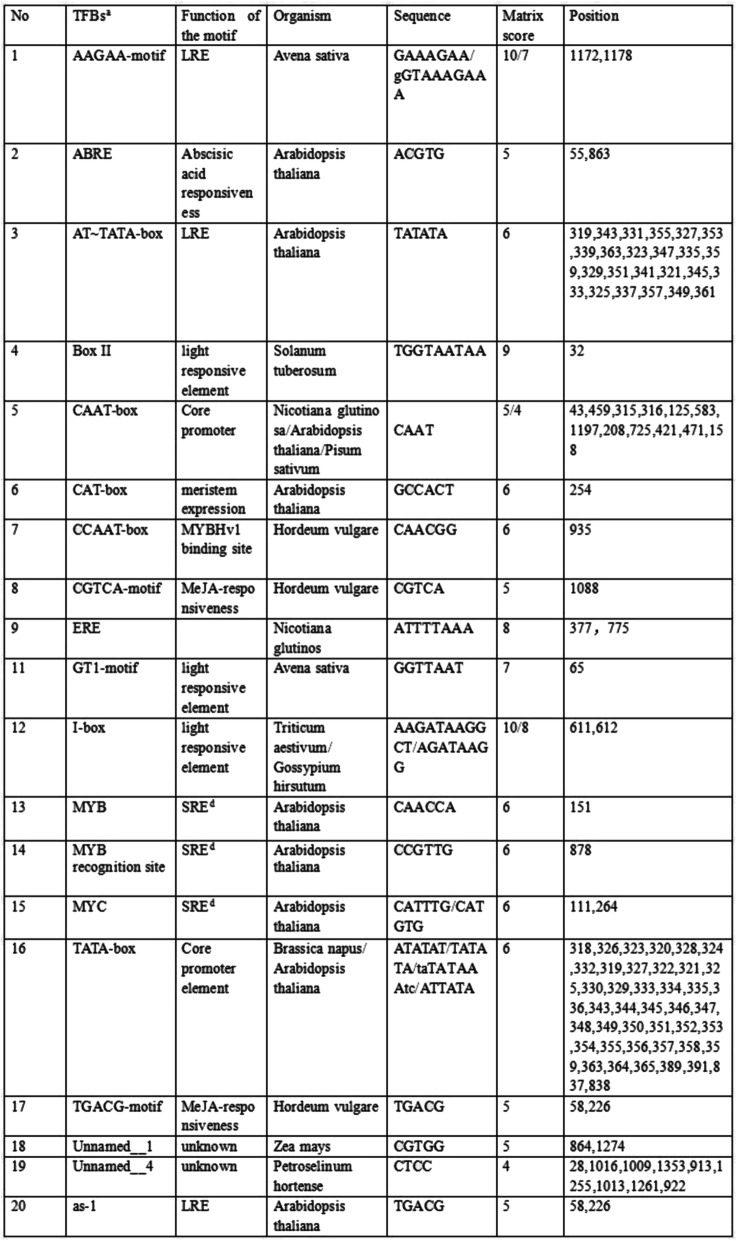
Known cis-acting elements in the pMaPIP1;1 using the PlantCARE and PLACE databases

### Activity of MaPIP1;1 promoter deletion mutants in transgenic *Arabidopsis thaliana*

**Fig. 2:**
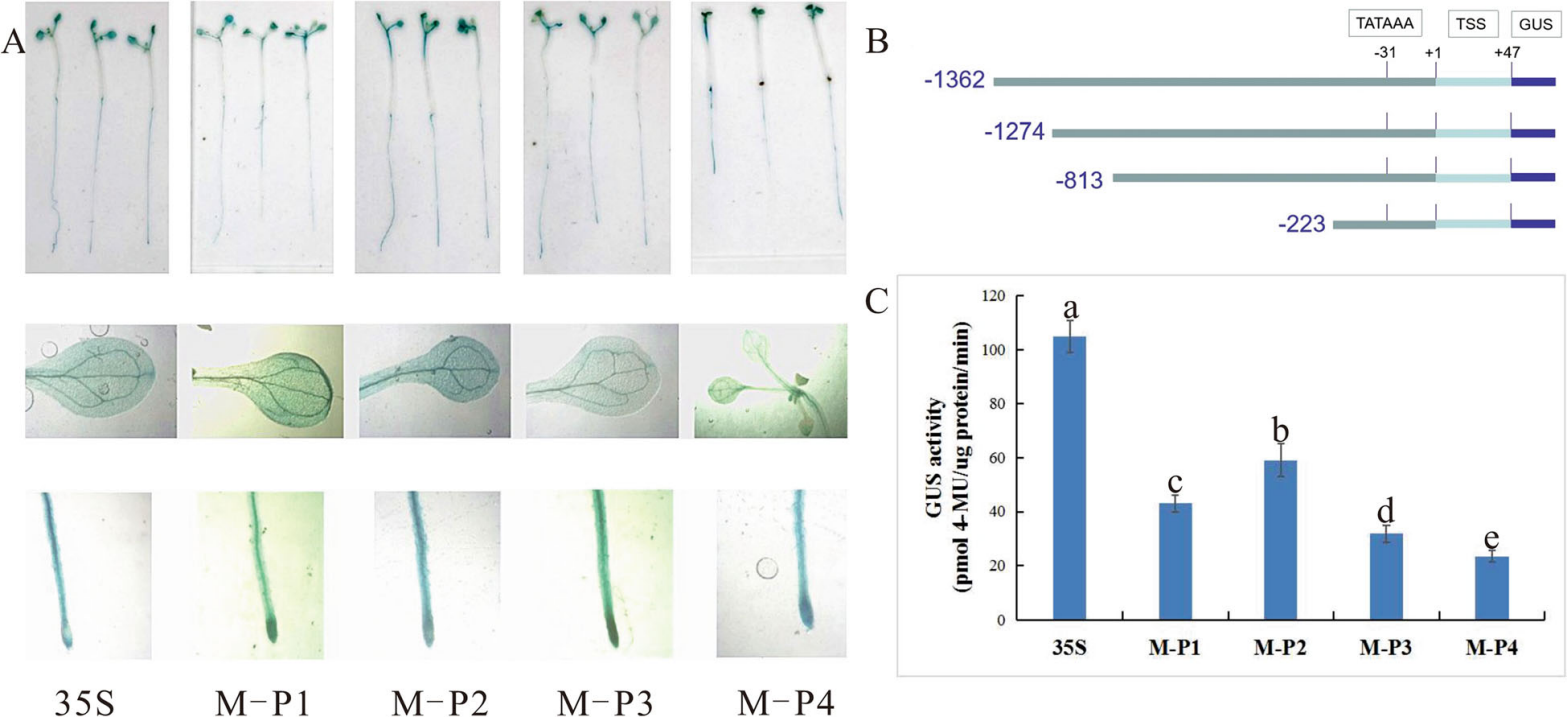
GUS activity in transgenic Arabidopsis. (**a**) Histochemical GUS staining in the different tissues. (**b**) The constructs of the truncated fragments of pMaPIP1;1 fused with GUS. TSS, transcription start site. (**c**) Fluorometric GUS assays of M-P1--M-P4 transgenic Arabidopsis in comparison with the positive control CaMV35S promoter. Error bars show standard deviation. GUS activity was measured in pmol 4MU/μg protein/min. Values represent the mean ± standard deviation from three independent transgenic lines and each line five individual plants for each construct. Different lower case letters above the bars indicate significant differences at *P* < 0.05

To characterize the MaPIP1;1 promoter containing sequences that are critical for drought stress responses, four fragments containing segments of the promoter region, termed M-P1, M-P2, M-P3, M-P4 that were 1362 bp, 1274 bp, 813 bp, 223 bp, respectively, were replaced 35S promoter of the pCAMBIA 1304 vector separately transformed into *Arabidopsis thaliana*. At least three independent T3 transgenic Arabidopsis lines were established for each deleted promoter construct to determine the promoter activity. GUS enzyme activity assays showed that M-P1 and M-P2 had higher activity than did M-P3 and M-P4. The GUS activity of M-P2 was up to 60 pmol 4MU/μg protein/min, which was 0.57-fold higher than the control, and 1.88 and 2.22 fold higher than that for the M-P3 and M-P4 fragments, respectively (Fig. 2c). And the GUS staining were consistent with the GUS enzyme activity, showed that M-P1-M-P4 could all drive GUS expression, but their promoter activities differed in transgenic *Arabidopsis thaliana*, we can clearly observe that M-P2 has the deepest root staining (Fig. 2a). These results indicate that M-P2 (− 1274 to − 1) is the core region of the MaPIP1;1 promoter, which contains many core region components as well as a CAAT-box and TATA-box.

### Drought stress responses mediated by four deletion mutants of the MaPIP1;1 promoter in transgenic *Arabidopsis thaliana*

**Fig. 3:**
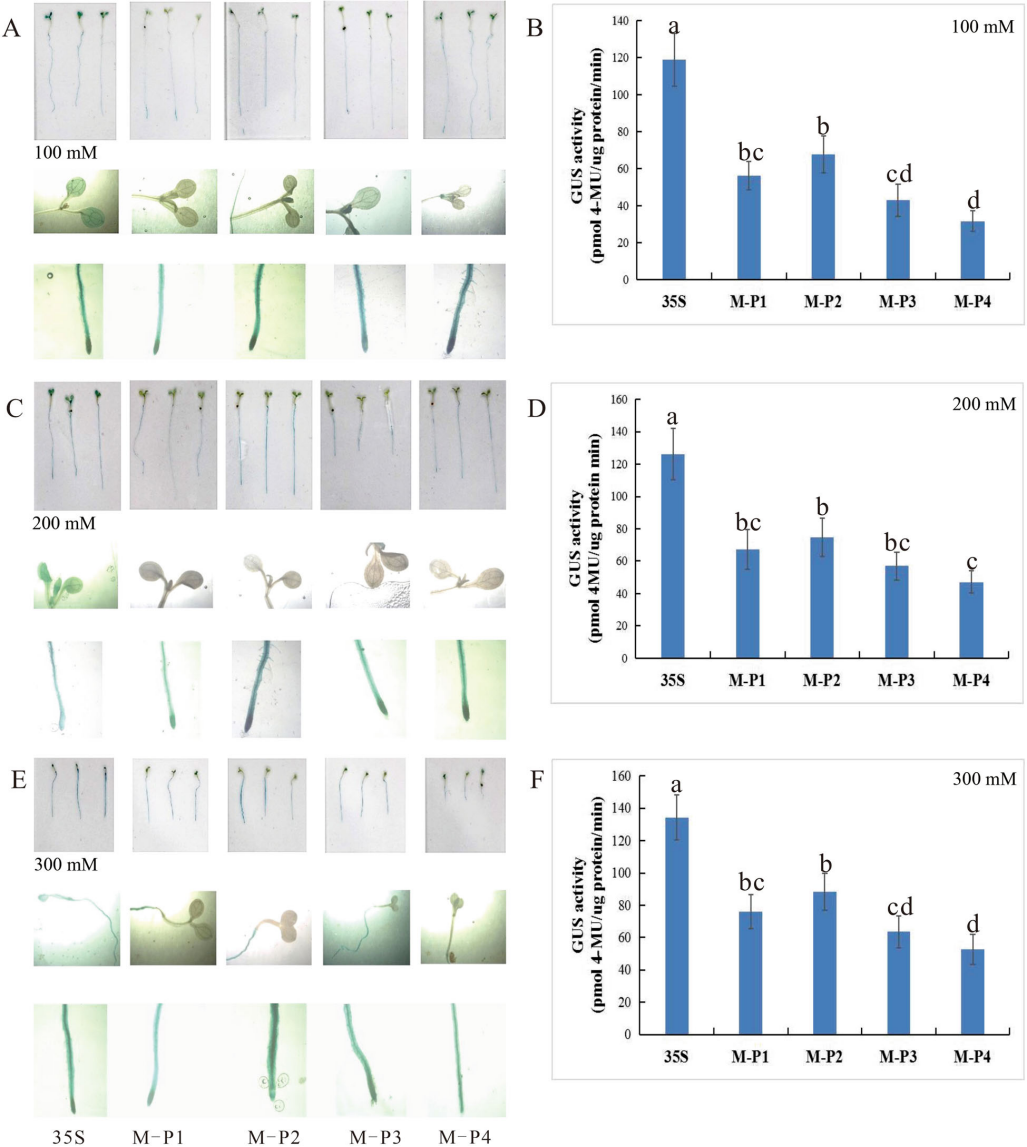
GUS histochemical staining and fluorescent quantitative analysis of transgenic Arabidopsis expressing four truncated promoter of pMaPIP1;1::GUS under drought stress conditions. (**a**, **c**, **e**) GUS histochemical staining of transgenic Arabidopsis with 100 mM, 200 mM, 300 mM mannitol respectively. (**b**, **d**, **f**) GUS fluorescent quantitative analysis of transgenic Arabidopsis with 100 mM, 200 mM, 300 mM mannitol respectively. Values represent the mean ± standard deviation from three independent transgenic lines and each line five individual plants for each construct. Different lower case letters above the bars indicate significant differences at *P* < 0.05

To further investigate the regulatory mechanism of MaPIP1;1-mediated response to drought stress, transgenic 15 day-old *Arabidopsis thaliana* seedings transformed with the deletion mutants or CaMV35S promoter as a control were treated with 100 mM, 200 mM or 300 mM mannitol. The GUS enzyme activity was more accurately determine the difference in GUS expression between the fragments under drought stress. Compared with the control, the GUS enzyme activity of each fragment in M-P1-M-P4 increased with increasing concentrations of mannitol. Plants transgenic for M-P2 (− 1274 to − 1) showed higher enzymatic activity. At 300 mM mannitol, the GUS enzyme activity for M-P2 was 89 pmol 4MU/μg protein/min, the control is 1.48-fold higher than that. This result indicates that functional elements related to drought stress may be contained between M-P2 and M-P3. The enzymatic activity in CaMV35S transgenic *Arabidopsis thaliana* was stable at each treatment concentration. The ratio of M-P2 and CaMV35S at 300 mM mannitol was higher than that at 0 mM mannitol, suggesting that M-P2 (− 1274 to − 1) can drive high levels of gene expression and drought stress induction (Fig. 3b, d, f). The GUS staining due to the promoter was detected separately from the leaves and roots. Under different concentrations of mannitol, GUS staining in the leaves of transgenic plants was either absent or faint in plants carrying M-P1-M-P4, but was present in the CaMV35S control plant leaves. In roots of the transgenic plants, the apical region was darker. Similar to the GUS enzyme activity results, the transgenic *Arabidopsis thaliana* carrying M-P2, encoding the segment between − 1274 and − 1, had the most intense staining, whereas that for M-P3 (− 813 to − 1) was lighter and the other fragments had similar staining intensity (Fig. 3a, c, e).

### Proteins bound to the drought-responsive promoter region

A yeast one-hybrid method was next used to verify that M-P2 contained the core region of MaPIP1;1 promoter and to characterize the molecular mechanism of MaPIP1;1 regulation in drought stress. M-P2 was used as the bait segment to construct the bait vector, and a drought-treated banana cDNA single-hybrid library in yeast was screened to identify proteins that interact with core region sequences.

**Fig. 4:**
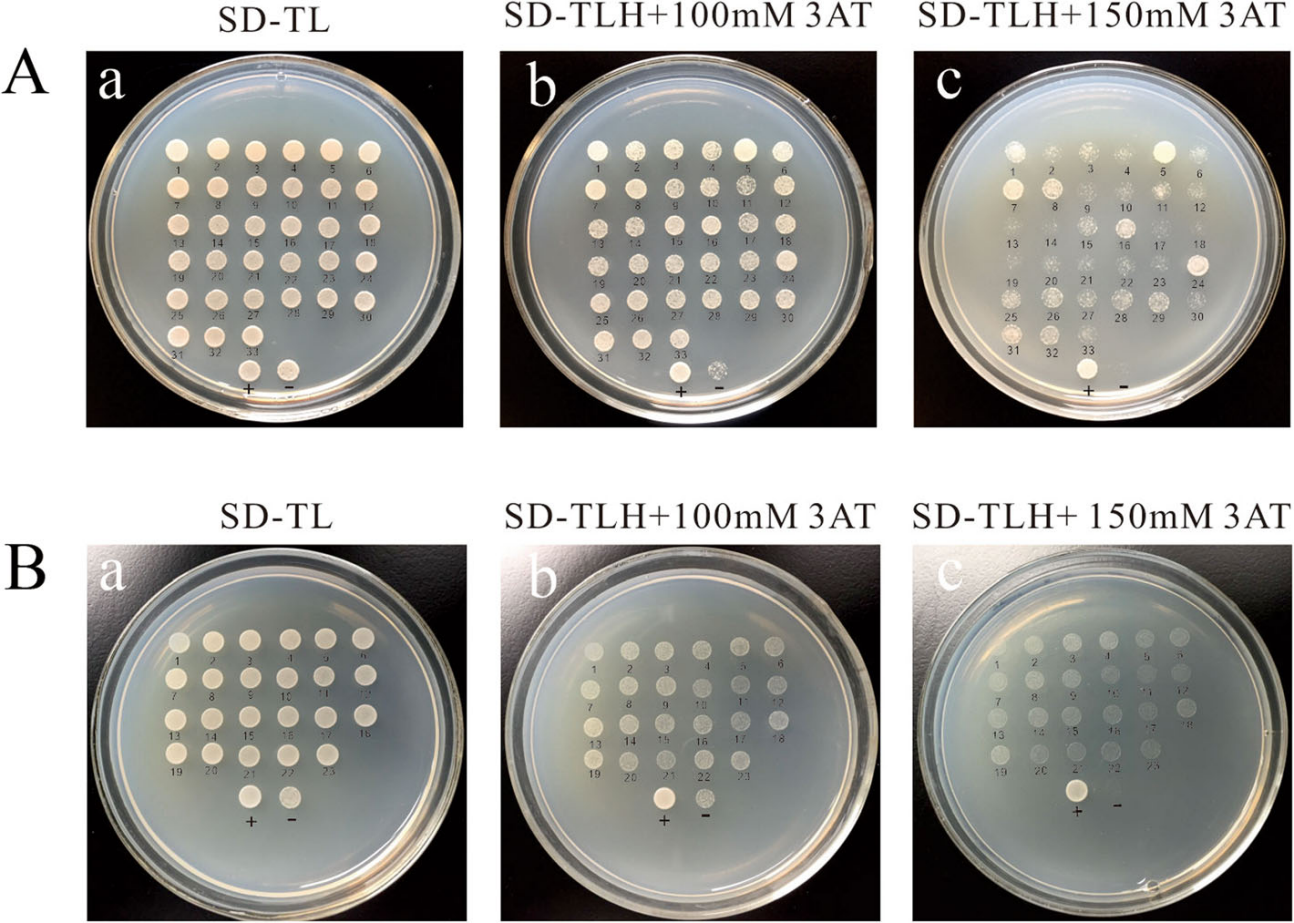
Identifification of proteins interacting with pMaPIP1;1 by a Y1H assay. (**A**) Detection of positive clones against HIS3 reporter gene activation. (a.b.c) Adding to 0 mM, 100 mM, 150 mM 3AT respectively. (**B**) Positive colony rotation verification of HIS3 reporter gene detection. (a.b.c) Adding to 0 mM, 100 mM, 150 mM 3AT respectively

The colonies were further tested on screening plates with 3-Amino-1,2,4-Triazol (3AT). The total number of transformants is 7.46 × 10^5^. A total of 29 colonies could activate the HIS3 reporter gene to varying degrees, and were subjected to DNA sequencing and BLAST alignment analysis to obtain 23 different proteins that interacted with the MaPIP1;1 promoter sequence. All 23 positive clones activated the HIS3 reporter gene (Fig. 4D) and carried genes for beta-amylase (BMY), acidic chitinase, pectate lyase 2 (PL2), neutral ceramidase, glucan endo-1,3-beta-glucosidase, V-type proton ATPase subunit d2, germin-like protein, KH domain-containing protein, heat shock cognate 70 kDa protein, 3′-N-debenzoyl-2′-deoxytaxol N-benzoyltransferase, tubulin alpha-3 chain, alpha-1,4 glucan phosphorylase L isozyme, pyrrolidone-carboxylate peptidase, ADP-ribosylation factor, pyruvate kinase, levodione reductase and MADS box protein (MADS3).

### qRT-PCR detection of binding proteins induced under drought stress

**Fig. 5:**
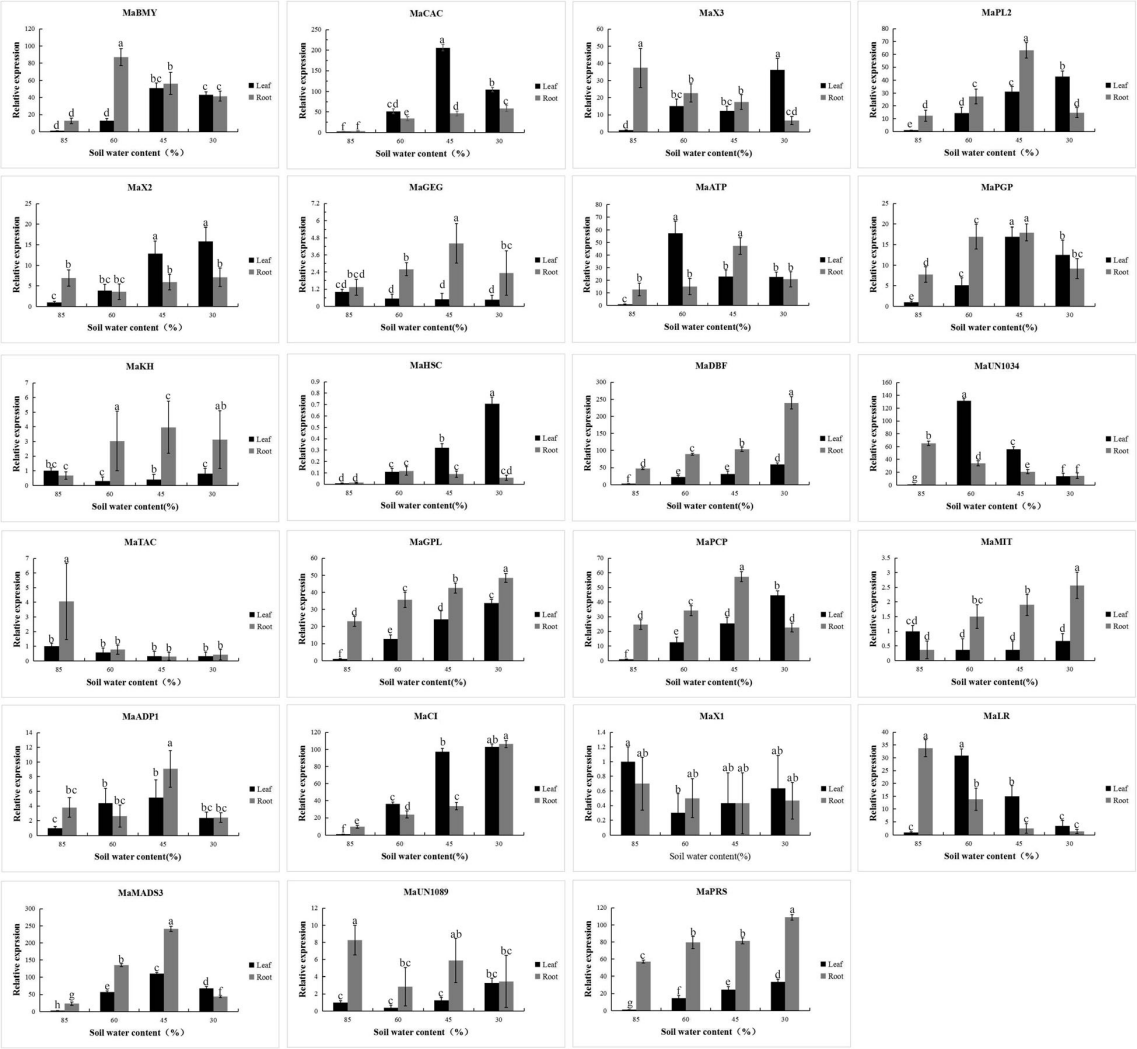
Expression analysis of 23 positive clones under drought stress of leave and root in the banana. The details shows in Table 1. Data are means ± SD of *n* = 3 biological replicates. Means denoted by the same letter are not significantly different at *P* < 0.05 as determined by Duncan’s multiple range test

The expression patterns of the 23 binding proteins in the leaves and roots of five-leaf and one-hearted banana seedlings under various drought stress conditions were screened by qRT-PCR (Fig. 5). In the leaves,the expression levels of six genes, MaBMY, MaCAC, MaATP, MaPGP, MaADP1, and MaMADS3, show increased and then decreased. The expression levels of ten genes, MaX3, MaPL2, MaX2, MaHSC, MaDBF, MaGPL, MaPLP, MaCI, MaUN1089,MaPRS show increased. And the expression levels of three genes, MaGEG, MaTAC, MaX1 was decreased,the expressions of MaKH and MaMIT have not changed significantly. In the root system, the expression of MaPL2, MaATP, MaPGP, MaPCP, MaADP1, MaMADS3, MaGEG, MaKH show increased and then decreased. The expression of MaBMY, MaCAC, MaDBF, MaGPL, MaCI, MaPRS, MaMI show increased. And the expression of MaX3, MaLR, MaUN1089, MaTAC show decreased, MaX2, MaHSC, MaX1 show no obvious trends in terms of expression. We focused on one of these proteins, the transcription factor MADS3, for its effects on gene expression patterns in leaves and roots of banana plants exposed to drought stress. The expression pattern of MaMADS3 under banana drought treatment is consistent with the expression pattern of MaPIP1; 1. MADS3 expression was induced by drought stress. In leaves of banana plants exposed to drought stress in the form of 45% soil moisture, the MADS3 gene showed the highest expression, 369, in the leaves. In roots, MADS3 gene expression under the same conditions was similar to that seen for leaves and was 532. This result further demonstrates that the MADS3 transcription factor can cooperate with the MaPIP1;1 gene in response to drought stress in banana plants.

### Activation of the MaPIP1;1 promoter by MaMADS3

**Fig. 6:**
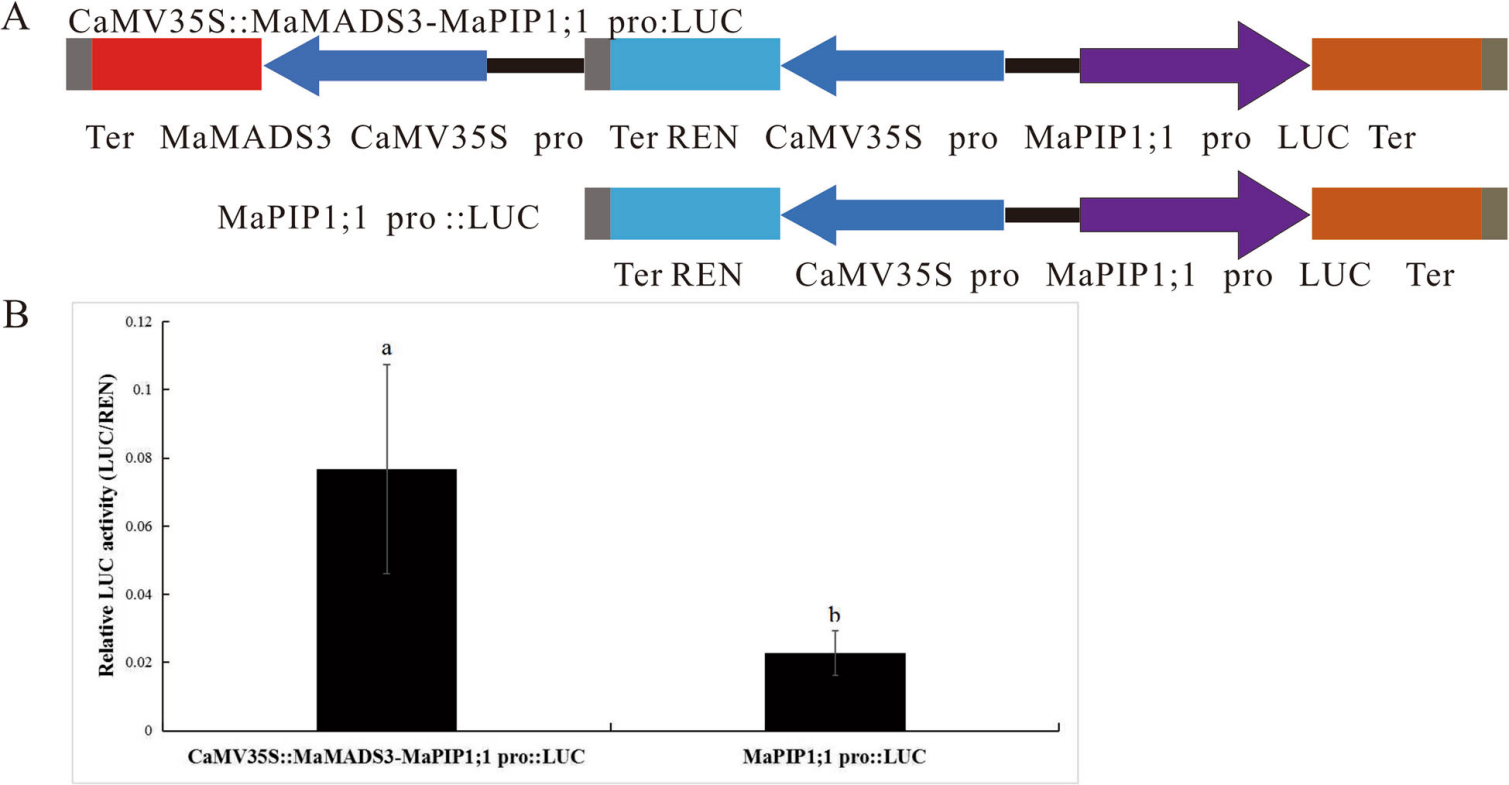
Activation of MaPIP1;1 promoter in the transient expression system by MaMADS3. (**A**) Schematic diagrams of the transient expression vectors used in the transient expression analysis. (**B**) Relative LUC activity of vector pCaMV35S::MaMADS3-MaPIP1;1 Pro::LUC and pMaPIP1;1 Pro::LUC in tobacco. Data are means ± SD of *n* = 3 biological replicates. Means denoted by the same letter are not significantly different at *P* < 0.05 as determined by Duncan’s multiple range test

To further demonstrate that MADS3 binds to the MaPIP1;1 promoter region, a dual luciferase assay was performed. The MaPIP1;1 promoter M-P2 fragment was cloned into a reporter vector pGreen II 0800 vector termed pMaPIP1;1::LUC, and the full-length ORF for MADS3 was cloned into the effector vector pCAMBIA 1301 vector to generate 35S::MADS3 (Fig. 6). These two constructs were transformed into GV3101 Agrobacterium, which were infiltrated to the far side of tobacco leaves for expression. The relative luciferase activity of pMaPIP1;1::LUC was lower than that of 35S::MADS3-pMaPIP1;1::LUC, suggesting that MADS3 can positively regulate MaPIP1;1 transcription in banana.

## Discussion

Abiotic stresses such as drought have caused significant losses in plant production worldwide [1]. Water is essential for plant production [2] and all processes involving water transport including drought adjustment, single cell expansion and long distance transport, require movement of water through cell membranes [3]. AQPs enhance water permeability in plant vacuoles and plasma membranes [4] and play important roles in the physiology of water balance and efficient water use [5–10]. Plants have numerous aquaporin homologs. The abundance and activity of AQPs in the plasma membrane is regulated in order to control water fuxes within the cells and in and out of the cells [15]. PIP proteins belonging to the AQP family function in water transport across the plasma membrane in multiple plant species [6–8]. Many PIP family proteins are involved in abiotic stress responses [13]. Results from this study demonstrated that MaPIP1;1 can improve plant drought resistance of transgenic Arabidopsis [22].

We characterized the MaPIP1;1 promoter to understand its function in response to drought stress. Analysis of four MaPIP1;1 deletion mutants, M-P1-M-P4, under drought stress conditions revealed that M-P2 carrying the sequence − 1274 to − 1 upstream of the transcription initiation site ATG is a key region of the MaPIP1;1 promoter that mediates the response to drought stress. Transient measurements of GUS enzyme activity in *Arabidopsis thaliana* also showed that M-P2 was sufficient to respond to drought stress. PLACE and PlantCARE applications indicated the presence of multiple TATA-boxes and one CAAT-box in the MaPIP1;1 promoter. The TATA box is a core promoter element for gene expression at the transcription initiation site. The CAAT-box is a common cis-acting element in promoters that have enhancer activity. However, no response elements related to drought stress were found in the MaPIP1;1 promoter. We have found the ABRE which is acis-acting element of abscisic acid (ABA). The studies have reported that ABA is an important plant hormone involved in many important biological processes and reactions, including abiotic stresses [41, 42], so we speculate that MADS3 may regulate the expression of this gene by binding to the ABRE element on the MaPIP1;1 promoter. Or maybe the M-P2 region may contain as yet unknown cis-acting elements that are critical for MaPIP1;1-mediated responses to drought stress. Nonetheless, the identification of cis-acting elements and interacting proteins in this promoter region highlight a potential important role for MaPIP1;1 in responses to drought stress.

Promoters are important for initiating gene transcription and regulate gene expression in both a temporal and spatial sense. Genetic modifications that target genes and their promoters can be used to enhance crop performance [43]. However, overexpression of transgenes can hamper plant growth and development, by imparting inefficient energy metabolism or morphological and/or physiological dysfunction [27–43]. Thus, inducible promoters that selectively drive expression of target genes under specific environmental conditions can be used to avoid negative effects of excess expression that can occur with the use of constitutive promoters [44–47]. From Fig. 2 we can see that, apart from the control 35S promoter, M-P2 is the darkest of the four fragments and has the highest GUS enzyme activity. This indicates that the promoter fragment can better respond to drought stress. And previous experiments showed that the MaPIP1; 1 gene can resist drought stress [30]. This further verified that the fragment can promote to the MaPIP1; 1 gene, and better resist drought stress. Some plants have stronger resistance to stress when transduced with stress-inducible promoters. For example, the truncated 219-bp fragment (D8) of the maize promoter ZmGAPP can confer high levels of gene expression under high saline conditions or in the presence of drought stress in transgenic tobacco [48]. Here we used deletion mutants to identify a 1274 bp (M-P2) segment as the core fragment of the MaPIP1;1 promoter that had the highest transcriptional activity among the mutants. In *Arabidopsis thaliana* transgenic for the M-P1-M-P4 constructs, the promoters had between 57 and 84% higher activity relative to the control CaMV35S promoter under normal and drought stress conditions, respectively. Compared with CaMV35S, M-P2 may enhance MaPIP1;1 expression in transgenic plants exposed to stress conditions. The promoter fragment of MaPIP1;1 from banana (monocot) will be useful for drought- resistance breeding in *Arabidopsis thaliana* (dicot). The transcriptional activity and expression pattern of promoters derived from different sources may also have obvious difference in dicot and monocot. In monocots, ubiquitin promoters are generally more capable of driving transgene expression than the CaMV35S [49–54]. Some ubiquitin promoter derived from monocots fails to direct transgene expression in dicot. The CaMV35S promoter showed more than 10% the transcriptional activity of Ubi1in tobacco protoplasts [51]. However, some promoters display strong transcriptional activity generally in both dicots and monocots. For example, a 0.3 kb AtTCTP promoter shows high transcriptional activity in Arabidopsis (dicot) and creeping bentgrass (monocot) [55]. Further characterization of transcriptional activity and expression patterns of MaPIP1;1 promoter in banana and evaluation of its application prospect in genetic improvement of monocot crops will also be meaningful.

We also used a heterologous banana-derived promoter to regulate GUS expression in Arabidopsis to confirm that M-P2 conferred high levels of expression in transgenic Arabidopsis. Staining for the reporter GUS in transgenic *Arabidopsis thaliana* under drought stress treatment mainly concentrated in the root system (Fig. 4A,C,E), which indicated that the MaPIP1;1 promoter may have root-specific promoter elements, although additional characterization is needed to confirm this possibility. Together these results lay the foundation for future applications of this promoter to direct expression of genes in root tissues.

Although many studies showed that AQP gene expression is affected by abiotic stress, there are no reports concerning transcription factors that regulate AQP expression in plants. Here we performed yeast one-hybrid (Y1H) screening and identified 23 proteins that could act as upstream regulators of MaPIP1;1 expression. qRT-PCR showed that the expression pattern of each gene is not exactly the same. Although it can be seen from the figure that some genes are up-regulated under drought stress. However,these genes are not all in response to drought stress both in the leaves and roots. And it is also mentioned that in our previously published paper [30], MaPIP1;1 was induced in both leaves and roots. The highest expression of MaPIP1;1 was when the soil moisture content is 45%.Therefore,we selected the MADS3 gene consistent with its expression pattern for further research.

The MADS-BOX is associated with responses of plants to stress conditions. Expression of the rice OsMADS31 gene is down-regulated under high salt conditions and can respond to high salt stress during rice germination [56]. LcMADS1 and LcMADS2 expression in Mianyang grass is significantly induced by cold-induced injury, whereas LcMADS9 expression is up-regulated by NaCl [57]. In canola, 13 BrMADS genes responded to cold stress in qPCR expression analysis. Moreover, 19 BrMADS genes responded to drought and salt stress [58]. Here we showed that MaMADS3 binds the MaPIP1;1 promoter to regulate gene expression. However, whether MaMADS3 regulates MaPIP1;1 gene expression in vivo in banana remains unclear. Further studies on the relationship between MaMADS3 and MaPIP1;1 in banana are thus necessary to fully elucidate the complex regulatory mechanism of MaPIP1;1 expression in response to drought stress.

## Conclusions

In summary, the 1362 bp upstream of the MaPIP1;1 translation initiation site, was isolated from the banana genome. Four segments named M-P1, M-P2, M-P3 and M-P4 have been transformed to Arabidopsis, respectively and reacted well to drought treatment. M-P2 (− 1274 bp to − 1) showed the highest transcriptional activity among all transgenic Arabidopsis tissues, indicating that M-P2 contains the core region of pMaPIP1;1. Using M-P2 as a yeast one-hybrid bait, 23 different transcription factors or genes that interacted with MaPIP1;1 were screened. And the transcription factor MADS3 positively regulated MaPIP1;1 transcription which was further using an dual luciferase assay in vitro interaction detection. The results indicate that MaPIP1;1 mediates the molecular mechanisms associated with banana drought response and will enhance our understanding of AQP gene expression regulation patterns.

## Methods

### Isolation of MaPIP1;1 promoter sequence

The ATG start codon of MaPIP1;1 was obtained from the Banana A genome website (http://banana-genome.cirad.fr/) based on banana genome sequencing (Nature, 2012, 488: 213–217). The sequence was amplified from banana genomic DNA by screening primers for pMaPIP1; 1 (Table 1) and confirmed by sequencing. Finally, a 1362 bp fragment upstream of the transcriptional start site (TSS) of MaPIP1;1 was obtained by polymerase chain reaction (PCR) amplification using the PZ1 primer (Table 1) and was considered to be the full-length promoter.

### Bioinformatic analysis of the promoter sequence

The 1362 bp MaPIP1;1 promoter cis-acting element was analyzed using PLACE (http://www.dna.affrc.go.jp/PLACE/signalup.html) and PlantCARE (http://bioinformatics.psb.ugent.be/

/webtools/plantcare/html). The analysis and location of the active components are shown.

### Construction of promoter: GUS fusion plasmids

Four differently-sized 5′-terminal deletion fragments (M-P1 - M-P4) (− 1362, − 1334, − 813, − 223 to − 1 bp) were amplified with corresponding primers (Table 1) and the PCR products were cloned into the pCAMBIA 1304 vector (Cambia, Australia) digested with BamHI/EcoRI. The construct was verified by restriction digestion analysis and sequencing. The resulting constructs were used for stable transformation of *Arabidopsis thaliana* (Colombia),which was obtained from the BiyuXu lab of CATAS. And YiXu has undertooked the formal identification. The pCAMBIA1304 vector containing the CaMV35S promoter was used as a positive control.

### *Arabidopsis thaliana* culture and genetic transformation

The pCAMBIA1304 vector and the vector carrying M-P1-M-P4 promoters were transformed into Agrobacterium, which was cultured for 10 h with shaking at 28 °C in YEB medium containing Kan and Rif antibiotics and added to an Arabidopsis transgenic infiltration solution (1/2 MS, 50 g/L). *Arabidopsis thaliana* in sucrose was inverted and inoculated into bacterial liquid, and transformed using the inflorescence dip method. Dried seeds were soaked in 75% ethanol + 0.02% Triton X-100 solution for 10 min. The ethanol was removed, the seeds were washed 2–3 times and blown onto sterile filter paper to facilitate air drying before spreading on selection medium (1/2MS, 1.5% Sucrose, 0.7% Agra, 25 μg/mL hygromycin B). After incubating for 3 days in the dark at 4 °C, the seeds were transferred to the culture room and grown at 23 °C. Ten days later, seedlings showing normal growth were transplanted. In the flower buds, the proportion of seedlings and lethal seedlings that can grow normally in the T1 generation were calculated. Seeds from the T2 generation with a ratio of 3:1 were transplanted into culture medium, and after a week of acclimatization, were watered with an appropriate amount of 1/2TM Arabidopsis nutrient solution every 5 days. When the seeds had germinated, the T2 generation were harvested for subsequent experimental analyses.

### Drought treatments

Drought stress analyses were performed on Arabidopsis seedlings. For drought stress analyses, 4 day-old seedlings CaMV35S promoter transgenic plants and the M-P1-M-P4 transgenic plans were transferred to MS medium supplemented with 100–300 mM mannitol for 15 days. GUS staining, GUS enzyme activity assays and photographing were performed separately.

For drought stress determination in banana plants, the soil water content was measured according to previously described procedures (TZS-1, TOP, Zhejiang, China) and water was removed from banana seedlings grown in soil. The soil moisture content associated with different stress levels was outlined by Hsiao (1973) [59]. Samples were taken at the indicated time.

### Histochemical and fluorometric determination of GUS enzyme activity

Fresh samples (small plants) were placed in X-Gluc solution (GBT, St. Louis, USA) [60] for histochemical. The tissues were placed in GUS staining solution containing 50 mM sodium phosphate (pH 7.0), 0.5 mM potassium ferricyanide, 0.5 mM potassium ferrocyanide, 10 mM EDTA, 0.1% Triton X-100 and 1 mM X-Gluc, then D1-D3 and D4-D9 fragments incubated at 37 °C for 24 h. Fluorometric determination of GUS enzyme activity using 4-methylumbelliferyl glucuronide.

### Isolation and its quantitative analysis (qPCR) of total RNA

Total RNA was extracted from drought-treated five-leaf and one-hearted banana seedlings. The RNA (1 μg) was used to prepare cDNA using the Superscript III reverse transcriptase system. cDNA with an oligo (dT) primer according to the instruction of the PrimeScript first-strand cDNA synthesis kit (Takara) The cDNA was diluted 6-fold and 1.5 μL was used as a template in each reaction. Expression analysis was performed using RT-qPCR as described above. The resulting data were normalized with ACTIN2 and the fold-change in expression levels was calculated relative to the respective controls.

### Statistical analysis

Statistical software SAS 9.2 was used to perform t-tests, analysis of variance and chi-square tests to determine significant differences between the means (P < 0.05). Each experiment included three replicates.

### Yeast one-hybrid (Y1H) screening

Y1H screens were carried out using the Matchmaker Gold One hybrid library screening system (Clontech, Cat. No. 630304). The bait sequence was cloned into the pAbAi pHIS2 vector, conferring resistance to 3AT (a cyclic depsipeptide antibiotic used as a yeast selection marker). The resulting pHIS2-Bait construct was then linearized and integrated into the genome of Y1HGold yeast by homologous recombination to generate a bait-specific reporter strain. The minimum inhibitory concentration of 3AT of the bait-specific reporter strain was determined. Competent cells were prepared using Y187 yeast transformants containing the bait plasmid as recipients. The library plasmid pGADT7 was transferred into it and screened on selection medium SD-Trp-Leu and SD-Trp-Leu-His + 50 mM 3AT.The positive colonies grown on selection medium SD-Trp-Leu were diluted with sterile water and spotted onto SD-Trp-Leu, SD-Trp-Leu-His + 100 mM 3AT and SD-Trp-Leu-His + 150 mM 3AT-deficient plates, were grown on 30 °C for 3–4 days. And then the screening of positive clones were for plasmid extraction and sequencing.

### Dual luciferase assays

Dual luciferase assays were carried out as described by [61]. In this study, LUC and REN were derived from pGreen II 0800-LUC. The MaPIP1;1 promoter obtained by PCR was ligated to LUC digested with SpeI to generate MaPIP1;1 pro::LUC. MaMADS3 CDS was fused to the CaMV35 promoter following SalI digestion and ligated to MaPIP1;1 pro::LUC to generate CaMV35S::MaMADS3-MaPIP1;1 pro::LUC. Both constructs were introduced into the *A. tumefaciens* strain BLA4404. When the OD600 reached 0.6–1.0, the culture was infiltrated to the far side of tobacco leaves. Total protein was extracted from the infected area after 3 days of culture. Fluorescence values for LUC and REN were measured with the Dual Luciferase Reporter Assay System according to the manufacturer’s instructions (Promega, United States). The value of LUC was normalized to that of REN. Due to the sensitivity of the experiment, 16 replicates were used per experiment and 8–10 replicates were retained for analysis.

## Supplementary information

**Additional file 1 Table S1.**Polymerase chain reaction (PCR) primers of the promoter used in the present study.

**Additional file 2 Table S2.**Self-activation testing(A)Self-activation testing of the bait.(a.b.c) Inhibition of bait carrier self-activation add to 0 mM, 25 mM, 50 mM 3AT respectively.d.e.f,Positive control, pGAD53m + p53HIS.(g.h.i) Negative control,pGAD53m + pHIS2.(B) The library screening efficiency.(a.b.c) The dilution of 10,100,1000 multiples respectively

**Additional file 3 Table S3.**The 23 protein binding with MaPIP1;1.

## Data Availability

All data generated or analysed during this study are included in this published article and its supplementary information files.

## Abbreviations

AbA: Aureobasidin A
MeJA: methyl jasmonate
AQP: Aquaporins
ABA: abscisic acid
