# Supplementary figures and images for: Identification of transcription factors interacting with a 1274 bp promoter of MaPIP1;1 which confers high-level gene expression and drought stress Inducibility in transgenic Arabidopsis thaliana
Source: BMC Plant Biol. 2020 Jun 16;20:278. doi: 10.1186/s12870-020-02472-7 (PMC7298759; doi:10.1186/s12870-020-02472-7)

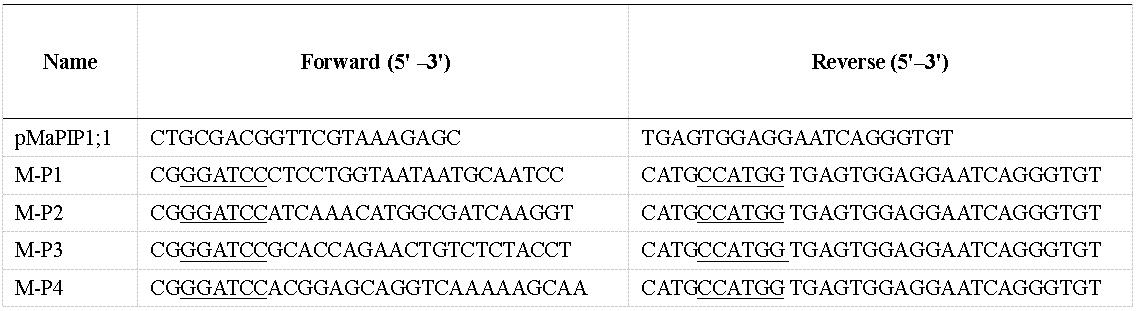

Supplement: Supplementary file 1 — Additional file 1 Table S1.Polymerase chain reaction (PCR) primers of the promoter used in the present study. [file 12870_2020_2472_MOESM1_ESM.jpg]

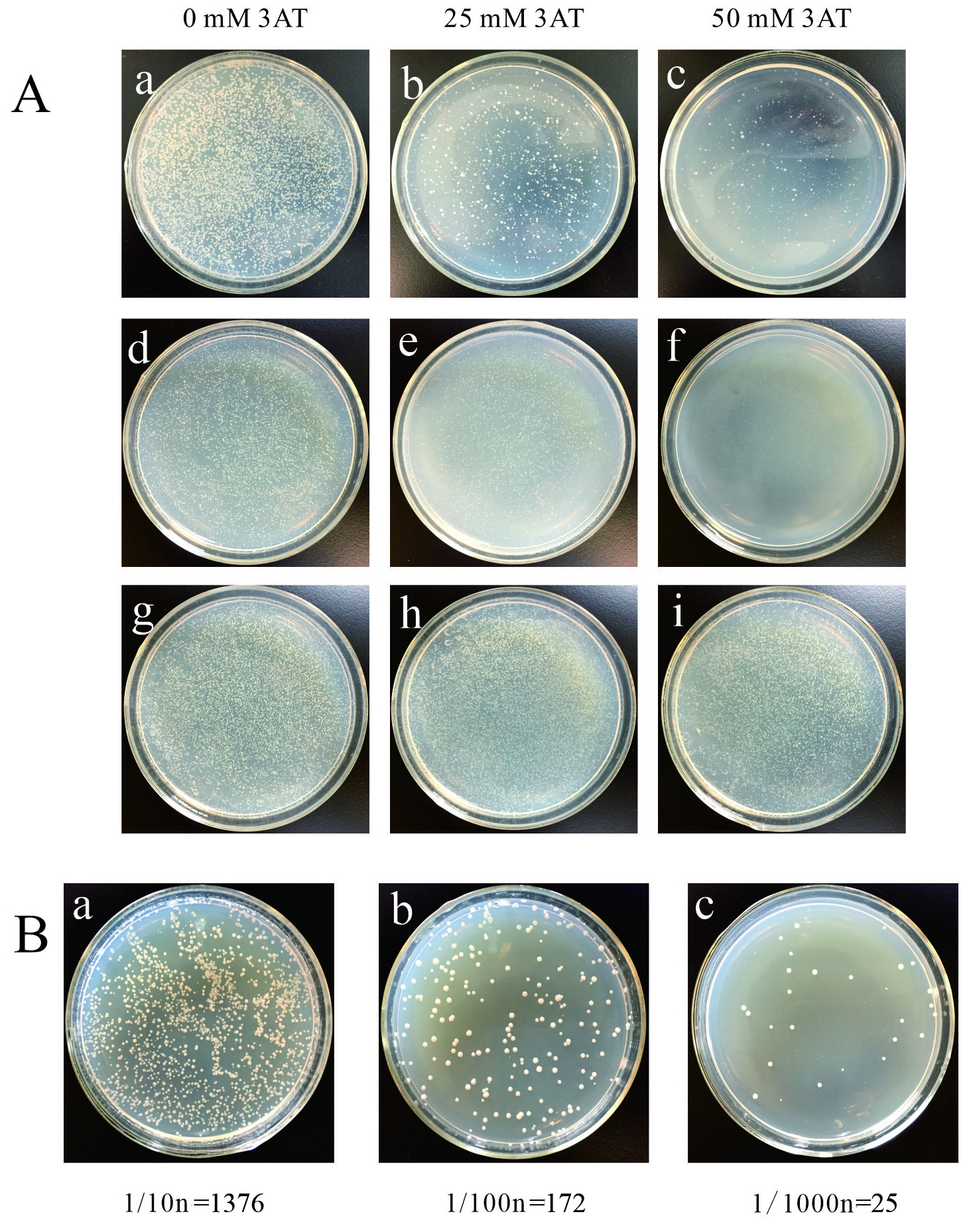

Supplement: Supplementary file 2 — Additional file 2 Table S2.Self-activation testing(A)Self-activation testing of the bait.(a.b.c) Inhibition of bait carrier self-activation add to 0 mM, 25 mM, 50 mM 3AT respectively.d.e.f,Positive control, pGAD53m + p53HIS.(g.h.i) Negative control,pGAD53m + pHIS2.(B) The library screening efficiency.(a.b.c) The dilution of 10,100,1000 multiples respectively [file 12870_2020_2472_MOESM2_ESM.jpg]

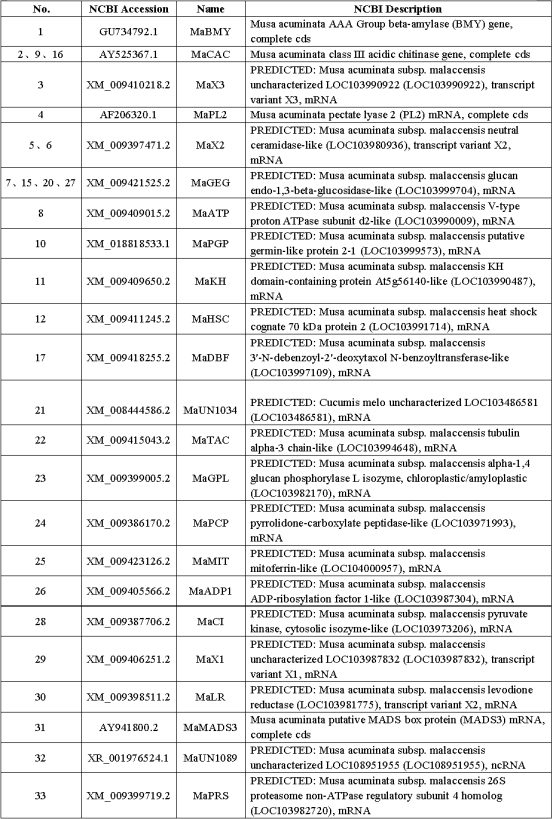

Supplement: Supplementary file 3 — Additional file 3 Table S3.The 23 protein binding with MaPIP1;1. [file 12870_2020_2472_MOESM3_ESM.jpg]
